# ﻿Discovery of a new hydrothermal copepod from the Indian Ocean and proposal of *Parabathyesola* gen. nov. (Harpacticoida, Laophontidae, Esolinae)

**DOI:** 10.3897/zookeys.1262.169224

**Published:** 2025-12-03

**Authors:** Jong Guk Kim, Il-Hoi Kim, Jimin Lee

**Affiliations:** 1 Division of Zoology, Honam National Institute of Biological Resources, Mokpo 58762, Republic of Korea Honam National Institute of Biological Resources Mokpo Republic of Korea; 2 Korea Institute of Coastal Ecology, Bucheon 14449, Republic of Korea Korea Institute of Coastal Ecology Bucheon Republic of Korea; 3 Marine Ecosystem Research Center, Korea Institute of Ocean Science & Technology, Busan 49111, Republic of Korea Korea Institute of Ocean Science & Technology Busan Republic of Korea

**Keywords:** Bathyal zone, hydrothermal vent, meiofauna, Onnuri Vent Field

## Abstract

Hydrothermal vent ecosystems are expected to harbor numerous meiobenthic animals; however, few studies have examined the species composition, abundance, and distribution of harpacticoid copepods in deep-sea hydrothermal assemblages. To gain insights into the natural biodiversity of such assemblages, we report a new species, *Parabathyesola calida***gen. et sp. nov.** (subfamily Esolinae, family Laophontidae), from a deep-sea hydrothermal vent in the Onnuri Vent Field located on the Central Indian Ridge. This species can be considered a sister taxon of the monotypic genus *Bathyesola* Huys & Lee, 2000, sharing six derived character states in the antennules, maxillulary endopod, maxillipedal syncoxa, and thoracic legs in both sexes. However, it differs from *Bathyesola* in the combination of a female antennule with six segments and the first endopodal segment in the third and fourth legs with an inner seta, justifying the establishment of a new monotypic genus, *Parabathyesola***gen. nov.** Therefore, the subfamily Esolinae now includes 19 species in nine genera. We also provide an amended dichotomous key to the genera of this subfamily.

## ﻿Introduction

Since the discovery of hot springs on the Galápagos Rift (Corliss et al. 1979), approximately 700 deep sea hydrothermal vents have been discovered on mid-ocean ridges, back-arc basins, and off-axis submarine volcanoes throughout oceans globally, of which more than 250 sites are active (Hannington et al. 2005; Beaulieu et al. 2013). Most vent sites that have been explored to date are in the Pacific and Atlantic oceans (Beaulieu et al. 2013, 2015; Wang et al. 2021), and little is known about the distribution of vent fields in the Indian Ocean (Kim et al. 2020). The Onnuri Vent Field located on the Central Indian Ridge (CIR) is one of 13 active vent sites known from the Indian Ocean (Kim et al. 2020; van der Most et al. 2023). The new vent is part of an ultramafic-hosted hydrothermal system with a low magma supply compared to other vents (e.g., Dodo and Edmond vent fields in the CIR), with high temperatures affected by magmatic activity (Kim et al. 2020; Lim et al. 2022). The hydrothermal environment of the Onnuri Vent Field is dominated by three bathymodiolus mussels (*Bathymodiolus marisindicus* Hashimoto, 2001; *Bathymodiolus* sp. 1; and *Gigantidas vrijenhoeki* Jang, Ho, Jun, Kim & Won, 2020), a stalked barnacle (*Neolepas marisindica* Watanabe, Chen & Chan, 2018), and 18 other microbenthic taxa (Jang et al. 2020; Kim et al. 2020). This vent site also harbors numerous meiobenthic taxa, including 65 unidentified nematodes (Kim et al. 2020) and ten valid copepods, including *Smacigastes pumila* Kim & Lee, 2020 (Harpacticoida), *Barathricola thermophilus* Ivanenko, Lee, Chang & Kim, 2019 (Cyclopoida), and *Aphotopontius limatulus* Humes, 1987, *Aphotopontius kiost* Lee, Kim & Kim, 2020, *Aphotopontius muricatus* Lee, Kim & Kim, 2020, *Benthoxynus constrictus* Lee, Kim & Kim, 2020, *Stygiopontius spinifer* Lee, Kim & Kim, 2020, *Stygiopontius horridus* Lee, Kim & Kim, 2020, *Stygiopontius geminus* Lee, Kim & Kim, 2020, and *Stygiopontius quadripaxillifer* Lee, Kim & Kim, 2020 (Siphonostomatoida) (Ivanenko et al. 2019; Kim and Lee 2020; Lee et al. 2020).

Despite the many previous studies conducted on hydrothermal vent areas, less attention has been paid to meiofauna, i.e., the microscopic benthic animals ranging in size from 30 to 1000 μm, than to mega- and macrofauna, and adequate sampling methods for this group have not been used (Heptner and Ivanenko 2002; Chertoprud and Novichkova 2023). The species diversity of meiofauna, including harpacticoid copepods, has rarely been studied in extreme environments (Willen 2003). To date, only 15 valid harpacticoid species have been characterized, in the families Aegisthidae Giesbrecht, 1893 (4 species), Tegastidae Sars, 1904 (3 species), Ancorabolidae Sars, 1909 (2 species), Tisbidae Stebbing, 1910 (2 species), Laophontidae Scott T., 1904 (2 species), Miraciidae Dana, 1846 (1 species), and Argestidae Por, 1986 (1 species) (Heptner and Ivanenko 2002; Willen 2004, 2006; Ivanenko and Defaye 2006; Plum and Martinez Arbizu 2009; Back et al. 2010); the two Tisbidae species parasitize cephalopods (Ivanenko and Defaye 2006). However, ecological studies have shown that cosmopolitan taxa such as Ameiridae, Ectinosomatidae, Miraciidae, and Thalestridae are also residents of vent habitats (Tsurumi and Tunnicliffe 2001; Diaz-Recio Lorenzo et al. 2021, 2023). A recent study on copepod communities in the Autonomous Benthic Explorer (ABE) hydrothermal vent field within the Lau Basin in the southwestern Pacific showed that species richness was higher in the *Bathymodiolus* habitats than in *Alviniconcha* and *Ifremeria* habitats (Diaz-Recio Lorenzo et al. 2021). The discovery of the Onnuri Vent Field by a research team at the Korea Institute of Ocean Science and Technology in 2018 provided an opportunity to explore harpacticoid species diversity within the *Bathymodiolus* habitat, leading to the identification of a previously undescribed species belonging to family Laophontidae.

Laophontidae is one of the most diverse harpacticoid families, including more than 320 species within 75 genera, and is subdivided into the subfamilies Esolinae Huys & Lee, 2000 and Laophontinae Scott T., 1904, with the latter containing 95% of the species within the family (Huys and Lee 2018; Fuentes-Reinés et al. 2021). Although most members of Laophontidae are common in various shallow-water benthic habitats, 14 species within four genera in the subfamily Laophontinae (*Laophonte* Philippi, 1840, *Cornylaophonte* Willen, 1996, *Weddellaophonte* Willen, 1996, and *Bathylaophonte* Lee & Huys, 1999) and four genera in the subfamily Esolinae (*Archesola* Huys & Lee, 2000, *Archilaophonte* Willen, 1995, *Bathyesola* Huys & Lee, 2000, *Esola* Edwards, 1891) have been recorded from deep-sea bathyal environments (i.e., depths of 200–4000 m) (Lee and Huys 1999; Huys and Lee 2000, 2018; Boxshall and Halsey 2004; Gómez and Rivera-Sánchez 2021). Among these, two *Bathylaophonte* species (Laophontinae) have been found from deep sea hydrothermal habitats: *Bathylaophonte azorica* Lee & Huys, 1999, southwest of the Azores on the Mid-Atlantic Ridge (Menez Gwen, Lucky Strike) and *Bathylaophonte pacifica* Lee & Huys, 1999, north of Easter Island on the East Pacific Rise (Lee and Huys 1999). Lee and Huys (1999) considered the constituents of this family to have been unsuccessful in colonizing deepwater habitats.

Despite efforts to investigate species diversity in the Onnuri Vent Field, many hydrothermal harpacticoids likely remain to be discovered in this region. Recently, a copepod in the subfamily Esolinae that could not be assigned to any of the eight known genera was collected from washings of a *Bathymodiolus* mussel community in the Onnuri Vent Field. In this study, we report a new genus and species, *Parabathyesola calida* gen. et sp. nov., with a detailed description and illustrations of both sexes, and discuss taxonomic aspects of the new species within the subfamily. This is the first record of the occurrence of harpacticoids belonging subfamily Esolinae in a hydrothermal ecosystem.

## ﻿Materials and methods

Hydrothermal fauna was investigated by using a video-guided hydraulic grab (Oktopus, Germany) during an oceanographic cruise (dive number GTV1806 in June 2018) of the KIOST at depths of 2,200 m in the Onnuri Vent Field. Macrofauna in the *Bathymodiolus* mussel bed was washed in freshwater and filtered through a 50-μm net to sample meiofaunal organisms. Harpacticoid specimens were sorted from the filtered sample and identified at familial level under a Leica M165C stereo microscope. Several specimens of the new species were transferred to glycerin and then dissected in lactic acid for a taxonomic process. Whole bodies and dissected appendages were prepared on a reverse slide (Humes and Gooding 1964) and examined under an Olympus BX50 microscope equipped with differential interference contrast (DIC) prisms. Pencil drawings of them were made using a drawing tube on the microscope and were digitalized with the aid of Adobe Photoshop® 2022 software. The type material of the new species was deposited in the Marine Biodiversity Institute of Korea (MABIK), Seochun, Republic of Korea and the other examined specimens were also kept in the Honam National Institute of Biological Resources (HNIBR), Mokpo, Republic of Korea.

For the description of *Parabathyesola calida* gen. et sp. nov., we followed the morphological terminology of Huys et al. (1996) and the setal armature formulae of swimming legs devised by Lang (1934). Abbreviations used in the text and figures are **ae**, aesthetasc; **BENP**, baseoendopod; **EXP(ENP)1(2, 3)**, first (second, third) exopodal (endopodal) segment of a thoracic ramus; **P1–P6**, first to sixth leg. Body length was measured from the anterior tip of rostrum to the posterior end of caudal rami in lateral view by the aid of the drawing tube. Scale bars in figures are given in μm.

## ﻿Systematic account

### ﻿Order Harpacticoida Sars, 1903

**Family Laophontidae Scott T., 1904**

**Subfamily Esolinae Huys & Lee, 2000**

#### ﻿Genus Parabathyesola gen. nov.

Taxon classificationAnimalia

CA3DA465-CF92-5E09-A8C7-66360E9A554D

https://zoobank.org/639EA965-C9DC-433F-816B-F35EEF78E304

##### Etymology.

The generic name alludes to the close relationship of the new genus with the genus *Bathyesola*. Gender: feminine.

##### Diagnosis.

Esolinae. Body subcylindrical, slightly depressed, with inconspicuous demarcation between prosome and urosome, usually flexible dorsally; integument on all body somites reticulated except for caudal rami. Rostrum prominent, defined at base, with one pair of dorsal sensilla. Cephalothoracic shield bell-shaped. Free pedigerous somites gradually tapering posteriorly. P5-bearing somite with remarkably boarder posterior half. Genital double-somite completely fused ventrally, with bilateral constriction and dorsal transverse suture. Genital double-somite and first free abdominal somite with posteriorly extended pleurotergites. Caudal rami distinctly separated from each other, elongate, with seven setae; set of setae I and II located at distal one-fourth of lateral margin; setae IV and V with fracture planes.

Antennule six-segmented, elongate, with aesthetasc on fourth and sixth segments in female; first segment with one small spinous outer projection; seven-segmented, subchirocerate in male, with geniculation between fifth and sixth segments, aesthetasc on fifth and seventh segments. Antenna with allobasis bearing one abexopodal seta; exopod one-segmented, with four setae. Mandible with well-developed coxa; gnathobase with several teeth and one serrate seta; palp two-segmented; basis with two distal setae; exopod represented by one seta; endopod with one lateral and two distal setae. Maxillule with well-developed arthrite, with one anterior seta; endopod one-segmented, with two distal setae. Maxilla with robust syncoxa bearing three endites; allobasis drawn out into stout claw; endopod very small with three setae. Maxilliped subchelate; syncoxa with two setae; claw-like endopod accompanying one small seta and one denticle.

P1–P4 with wide and narrow intercoxal sclerites. P1 with elongate protopods; exopod three-segmented, extending midlength of ENP1; EXP1 and EXP2 with outer spine; EXP3 with two outer spines and two geniculate distal setae; endopod prehensile, two-segmented; ENP1 elongate, unarmed; ENP2 small, with one claw and one delicate seta. P2–P4 with rectangular coxae, with long outer setae; exopods longer than endopods, three-segmented; endopods two-segmented; P3 endopod in male three-segmented, with separate and recurved outer spine instead of spinous apophysis. Armature formulae:

**Table:** 

	Exopod	Endopod
P2	0.1.123	1.221
P3	0.1.123	1.321 [1.1.220 in male]
P4	0.1.123	1.221

P5 two-segmented; baseoendopod elongate, with outer setophore bearing seta; left and right BENP fused to sclerite in male; endopodal lobe well-developed, with five setae in female, undeveloped and unarmed in male; exopod elongate, with six setae in female and five setae in male; proximal two outer setae on exopod of female displaced in same insertion position.

P6 represented by two small setae on both sides of genital operculum in female; asymmetrical in male, with one long seta on each side.

#### ﻿ Parabathyesola calida

Taxon classificationAnimalia

gen. et sp. nov.

EB262842-5539-50D7-B439-D8C3908697F3

https://zoobank.org/99B326CC-9A51-4A43-B7F8-D1C3C26F53F5

**Figure 1.**
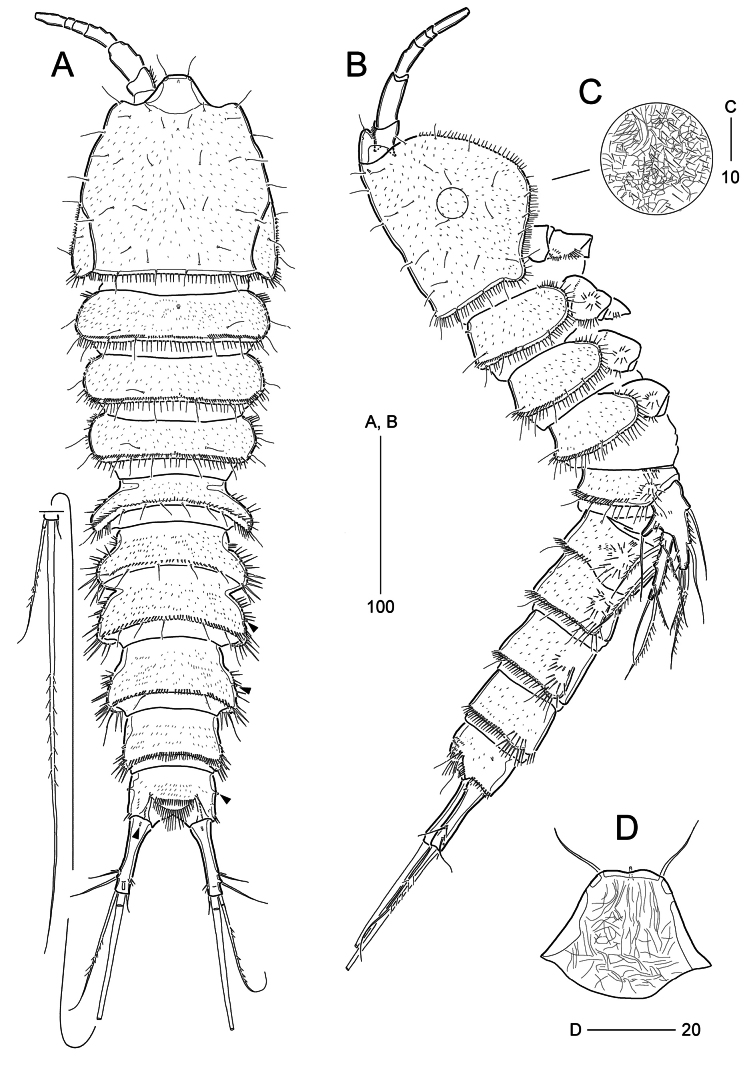
*Parabathyesola calida* gen. et sp. nov., female (MABIK CR00259487). A. Habitus, dorsal; B. Habitus, lateral; C. Integument of cephalothorax; D. Rostrum, dorsal. Arrowed points indicate tube pores on the genital double-somite, free abdominal somites, and caudal rami, respectively.

**Figure 2.**
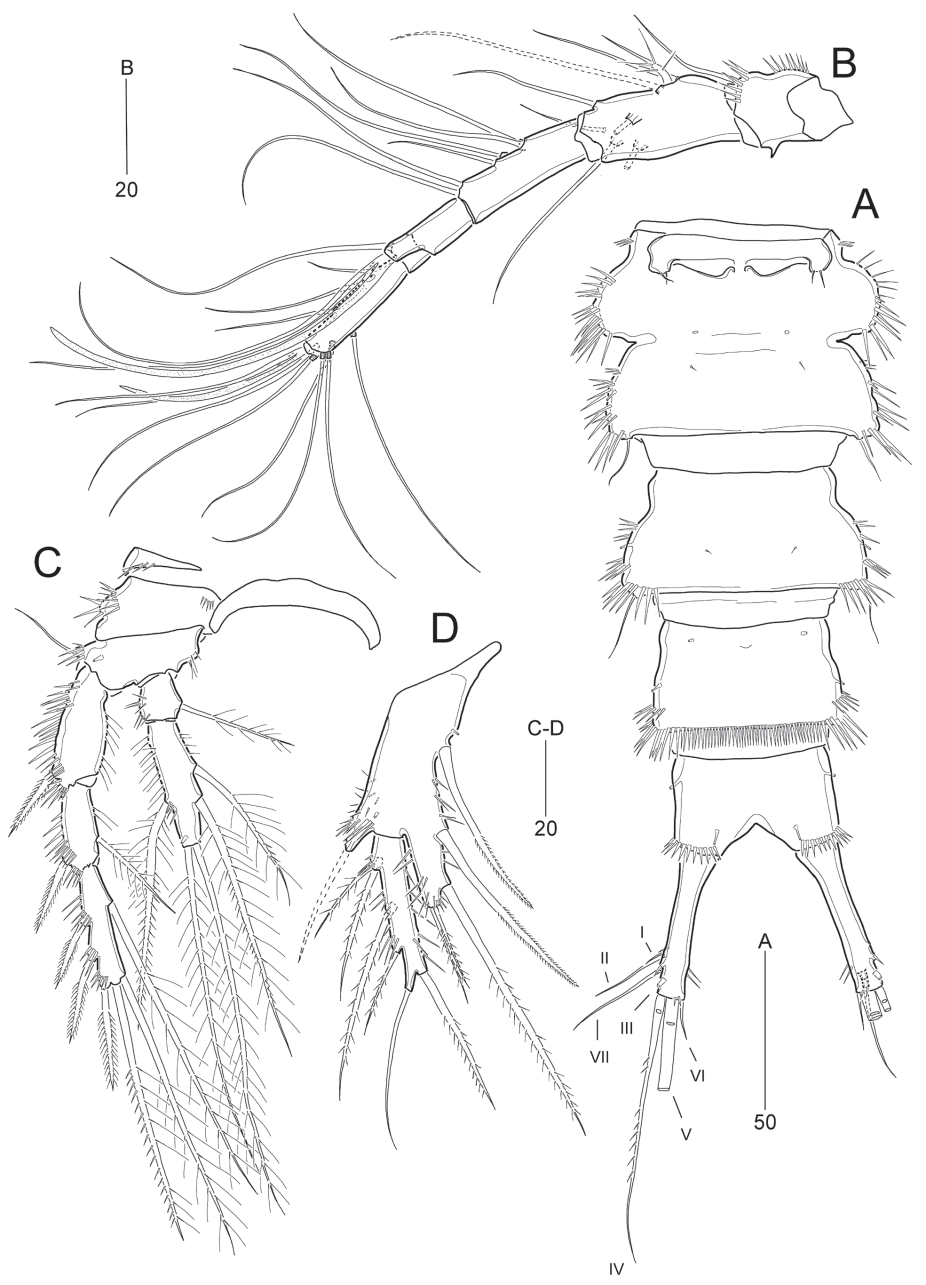
*Parabathyesola calida* gen. et sp. nov., female (MABIK CR00259489). A. Urosome excluding P5 bearing-somite, ventral; B. Antennule; C. P4; D. P5.

**Figure 3.**
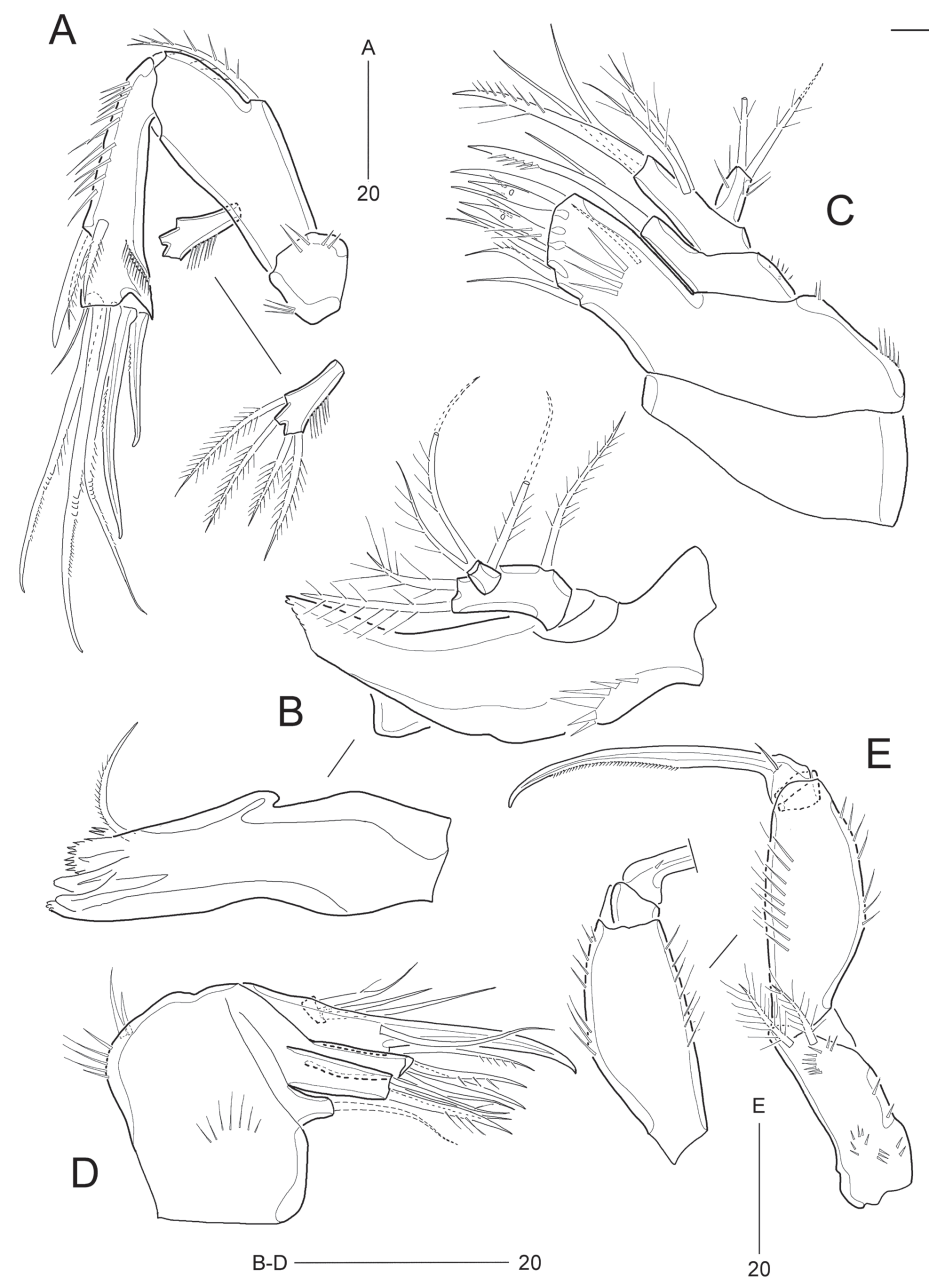
*Parabathyesola calida* gen. et sp. nov., female (MABIK CR00259489). A. Antenna; B. Mandible; C. Maxillule; D. Maxilla; E. Maxilliped.

**Figure 4.**
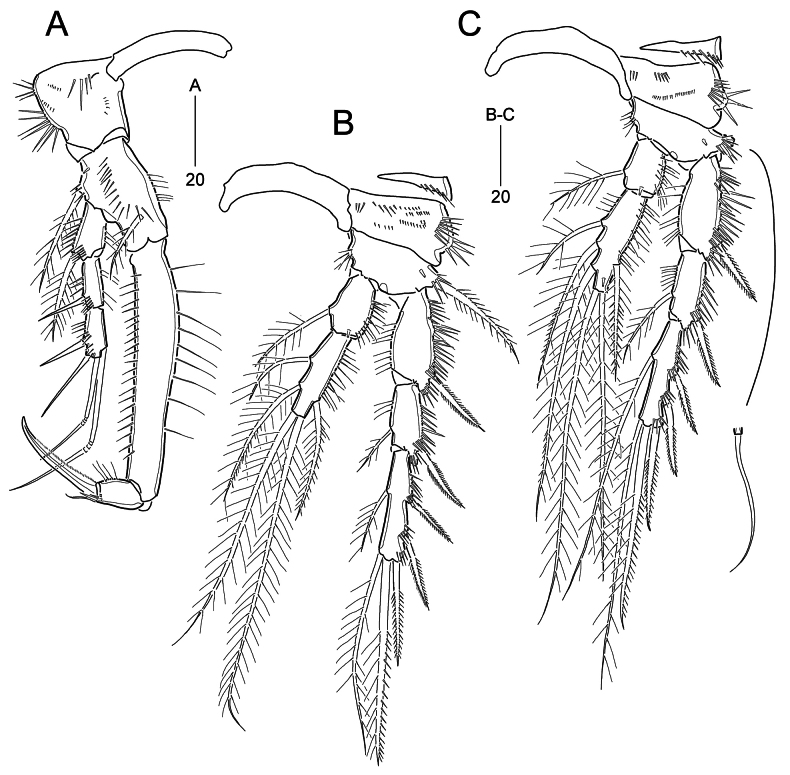
*Parabathyesola calida* gen. et sp. nov., female (MABIK CR00259489). A. P1; B. P2; C. P3.

**Figure 5.**
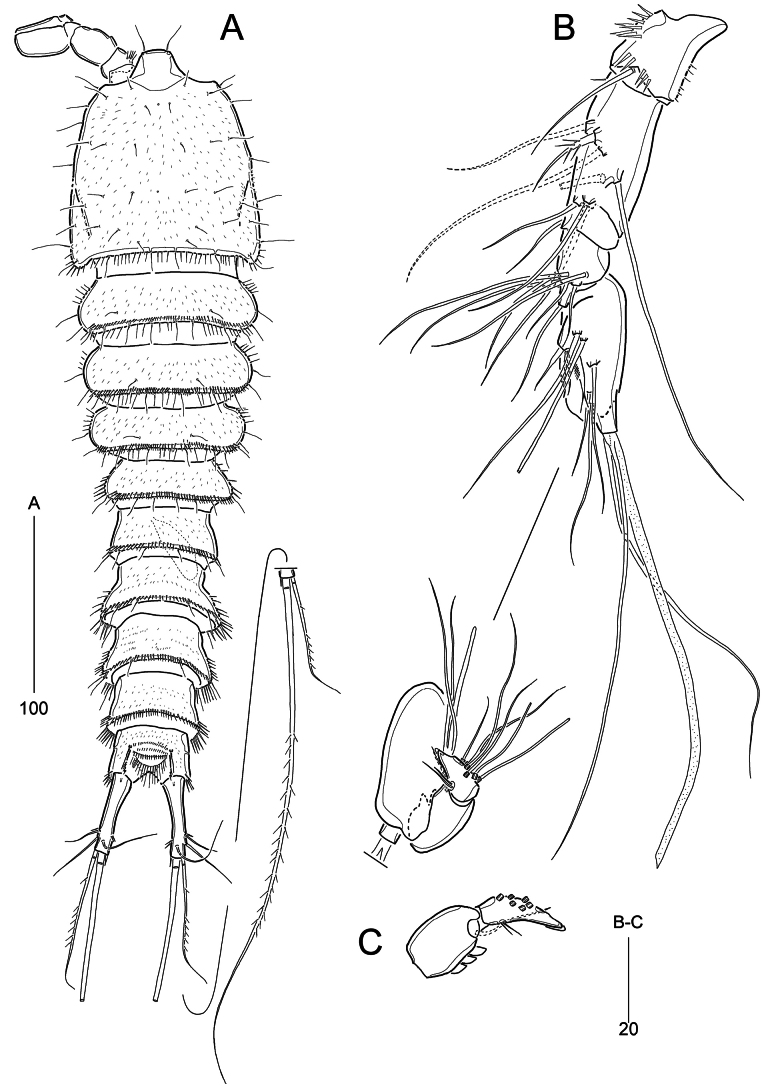
*Parabathyesola calida* gen. et sp. nov., male (MABIK CR00259490). A. Habitus, dorsal; B. Antennule; C. Distal two segments of antennule.

**Figure 6.**
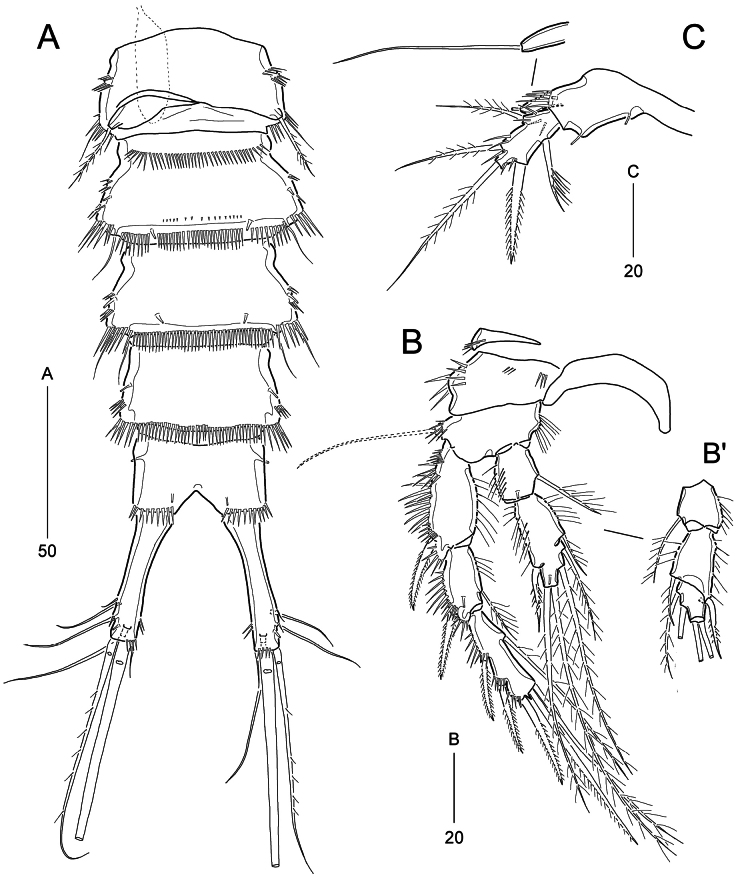
*Parabathyesola calida* gen. et sp. nov., male (MABIK CR00259490). A. Urosome excluding P5 bearing-somite, ventral; B. P3, B’. Endopod of P3; C. P5.

Figs 1, 2, 3, 4, 5, 6

##### Type material.

***Holotype*.** • intact female preserved in alcohol (MABIK CR00259486), Indian Ocean, Onnuri Vent Field located on the Central Indian Ridge (CIR), 11°24'52.99"N, 66°25'25.45"E, depth 2018.6 m; 24 June 2018, J. Lee leg. ***Paratypes*.** • one intact female (MABIK CR00259487), (whose antennules lost after drawing of habitus) and one intact male (MABIK CR00259488) preserved in alcohol, dissected and figured female (MABIK CR00259489) and male (MABIK CR00259490) mounted onto three slides, respectively. Five dissected females (MABIK CR00259491–CR00259495) and three dissected males (MABIK CR00259496–CR00259498) mounted onto a slide, respectively. Other intact two females and two males preserved together in alcohol (HNIBRIV25084). Sampling data of all paratypes as for the holotype.

##### Description.

**Female** (based on paratypes MABIK CR00259487 and CR00259489). Total body length ranged from 457–519 μm (mean = 490.1 μm, *n* = 7), excluding length of caudal setae. Body (Fig. 1A, B) subcylindrical, slightly tapering posteriorly, approximately 4× as long as wide; maximum width ~130 μm long measured at posterior end of cephalothoracic shield in dorsal view; demarcation between prosome and urosome inconspicuous, but flexible dorsally. Integument on all body somites reticulated (Fig. 1C), covered with fine spinules.

***Rostrum*** (Fig. 1D) well-developed, bell-shaped, defined at base, subapically with one mid-ventral pore and one pair of lateral pores; apical tip blunt, with two sensilla.

***Prosome*** (Fig. 1A, B) composed of cephalothorax and three free pedigerous somites (bearing P2–P4); first pedigerous somite completely incorporated into cephalosome. Cephalothoracic shield bell-shaped, ~1.2× as wide as long, with one mid-dorsal pore, and one unpaired sensillum (in central) and several paired sensilla; pleural areas rounded, with lobate posterior angles; posterior and ventro-lateral margins fringed with long fine setules. Free pedigerous somites gradually tapering posteriorly, with one dorsal row of fine spinules near posterior margin, one pair of dorsal sensilla, and five (in P2-, P4-bearing somites) or six (in P3-bearing somites) pairs of posterior sensilla; posterior and ventro-lateral margins fringed with long spinules; P2-bearing somite with one mid-dorsal pore.

***Urosome*** (Fig. 1A, B) five-segmented, comprising P5-bearing somite, genital double-somite, three free abdominal somites. All urosomites except for anal somite ornamented with one row of dorsal spinules posteriorly and two or three groups of long spinules laterally. P5-bearing somite with remarkably boarder posterior half and dorsal posterior margin ornamented with four pairs of sensilla and one pair of rows of long spinules. Genital double-somite completely fused ventrally (Fig. 2A), but original division marked by bilateral constriction and dorsal transverse suture, 0.77× as long as wide; anterior somite (genital somite) with border posterior half bearing three pairs of dorsal posterior sensilla; posterior somite gradually bordering towards posterior margin, with one pair of lateral tube pores (arrowed in Fig. 1A) and four pairs of dorsal posterior sensilla; genital gonopores and minute mid-copulatory pore covered by single opercula derived from sixth legs; P6 represented by two small setae on small lobes; midventral surface with one pair of pores and one pair of small sensilla. First free abdominal somite (fourth urosomite) comprising narrower anterior one-third and broadened posterior two-thirds, with one pair of lateral tube pores (arrowed in Fig. 1A) and three pairs of dorsal posterior sensilla. Second free abdominal somite (fifth urosomite) with almost straight lateral margins without sensillum and tube pore ornamentation. Anal somite smaller than preceding somite, with one pair of lateral tube pores (arrowed in Fig. 1A), one pair of ventral tube pores (arrowed in Fig. 1A), ornamented by posterior spinules laterally and ventrally; median cleft deep, ornamented by rows of lateral spinules on each side; semi-circular operculum ornamented with two rows of spinules, flanked by two sensilla. Caudal rami distinctly separated from each other, cylindrical, slightly swollen proximally, 3.4× as long as wide, ~1.4× longer than preceding somite, with one dorsal tube pore proximally and one row of inner spinules subdistally; set of setae I and II arising from distal one-fourth of lateral margin, seta I very small and seta II 4× as long as seta I; ventro-lateral seta III subdistal, posterior to set of setae I and II, ~1.6× as long as seta II; setae IV and V bi-serrate, with fracture plane, seta IV ~ 1.7× as long as ramus, seta V as long as urosome (including caudal rami); seta VI issuing at inner distal corner, small; dorsal seta VII arising from insertion level of seta III, longer than seta II.

***Antennule*** (Fig. 2B) six-segmented, elongate. First segment with one small spinous projection on outer margin and two rows of inner spinules. Second segment longest. Third segment slightly shorter than preceding segment. Fourth segment with distal pedestal bearing aesthetasc fused to adjacent seta. Fifth segment smallest. Sixth segment shorter than third segment, with acrothek composed of one aesthetasc and two setae. Armature formula as follows: 1-[1], 2-[8], 3-[6], 4-[1 + (1 + ae)], 5-[1], 6-[9 + acrothek]. All setae bare except for one spinulose seta on second segment; five outer setae on sixth segment bi-articulate basally.

***Antenna*** (Fig. 3A) composed of coxa, allobasis, one-segmented endopod, and one-segmented exopod. Coxa small, ornamented with two rows of spinules. Allobasis largest, 2.1× as long as wide, with one spinulose abexopodal seta. Free endopodal segment, longest, gradually broadening towards distal end; inner margin armed with long spinules proximal two-thirds, with one stout spine and two serrated setae; outer margin with surface frill composed of spinules. Distal armature comprising two spines, three geniculate setae, and one delicate seta; longest geniculate seta fused basally to delicate seta at inner distal corner. Exopod small, gradually broadening towards distal end, armed with one row of lateral spinules, with four plumose setae.

***Mandible*** (Fig. 3B). Coxa well-developed, with one row of stout spinules, one blunt median protrusion; gnathobase comprising one uni-cuspidate, two bi-cuspidate, one multicuspid teeth, and one uni-serrate seta. Palp composed of broad basis and one-segmented endopod; basis with two plumose setae distally and one plumose seta proximally (representing exopod); endopod small, with one lateral and two distal plumose setae.

***Maxillule*** (Fig. 3C). Parecoxa broad, with two rows of spinules on outer margin; arthrite well-developed, armed with one bare anterior seta and four posterior spinules; with two bare and two spinulose spines, one pinnate and three bare setae. Coxa small, armed with several outer spinules; endite cylindrical, with one bare seta and one spinulose spine. Base transversally elongate with two plumose setae laterally and one spinulose spine and two bare setae distally. Endopod small, 1.7× longer than wide, ornamented with lateral spinules, with two plumose setae distally.

***Maxilla*** (Fig. 3D). Syncoxa robust, ornamented with two stout spinules and one row of spinules on outer margin, one row of setules on surface, with three endites; proximal endite smallest, with one bare seta; middle endite largest, with one bare seta and two spinulose setae; distal endite, as large as middle endite, with one spinulose seta and two bare setae. Allobasis drawn out into stout claw accompanying one bare and one pinnate seta. Endopod very small, with one weakly plumose seta and two bare setae.

***Maxilliped*** (Fig. 3E) subchelate, composed of syncoxa, basis and endopod. Syncoxa ornamented with two groups of spinules and one row of spinules on surface, one row of spinules near outer margin, with two plumose setae distally. Basis largest, 2.4× as long as wide; outer margin convex, with two rows of long spinules, inner (palmar) margin almost straight, with two rows of long spinules. Endopod one-segmented, claw-like, uni-serrated, accompanying one small seta and one small denticle.

***P1*** (Fig. 4A). Praecoxa unexamined. Intercoxal sclerite transversely elongate, wide, slightly arched, without ornamentation. Coxa large, slightly longer than greatest width; outer margin slightly swollen proximal two-thirds bearing two groups of long spinules; anterior surface with one row of stout spinules and three rows of minute denticles. Basis elongated by development of pedestal for insertion of endopod, longer than coxa, ~1.7× as long as wide, ornamented with one row of inner spinules, one row of anterior spinules and one anterior tube pore; long and plumose outer seta issuing from pedestal positioned proximal one-fourth of outer margin and accompanying several anterior spinules; plumose inner seta shorter than outer seta, arising from distal one-fourth of anterior surface. Exopod 3-segmented, slightly less than midlength of ENP1; all segments subequal in length, ornamented with two rows of outer spinules and several inner setules (absent in EXP3); EXP1 with one long and plumose outer seta slightly beyond midlength of EXP3; EXP2 with one bare outer spine; EXP3 with two bare outer spines and two geniculate distal setae. Endopod prehensile, 2-segmented; ENP1 robust, elongate, ~5.5× as long as wide, ornamented with outer spinules and inner setules; ENP2 small, ornamented with outer setules and inner spinules (gradually increasing towards inwardly), distally with one delicate seta and one stout and uni-serrate spine.

***P2*** (Fig. 4B). Praecoxal small, ornamented with distal spinules. Intercoxal sclerite wide, arched, unornamented. Coxa large, ornamented with two rows of outer spinules and four rows of anterior spinules. Basis smaller than coxa, with one anterior tube pore near outer margin; inner margin ornamented with one group of setules and outer margin ornamented with one row of spinules; outer seta long and pinnate. Exopod longer than endopod, 3-segmented; EXP1 ornamented with five rows of outer spinules and one row of inner setules, with one pinnate outer spine; EXP2 smallest, with three groups of outer spinules and one row of inner setules, with one pinnate outer spine and one inner plumose seta; EXP3 as long as EXP1, ornamented with four rows of outer spinules, with three pinnate outer spines, one distal spine (ornamented with outer spinules and inner setules), one plumose distal seta, and one plumose inner seta. Endopod reaching distal one-third of EXP2; ENP1 slightly beyond midlength of EXP1, ornamented with outer setules, with one anterior tube pore and one plumose inner seta; ENP2 ~1.5× as long as ENP1, ornamented with outer setules, with one outer seta, two distal setae, and two inner setae; outer seta and distal inner seta with additional spinular ornamentation.

***P3*** (Fig. 4C) as in P2 except for setal armatures and ornamentations. Coxa with two rows of anterior spinules. Basis with one bare outer seta. Exopod same as setal armature of P2, but inner seta on EXP3 extremely long, 2.4× as long as EXP3. Endopod also slightly less than distal end of EXP2; ENP2 ~2× as long as ENP1; ENP2 with one anterior pore and inner setules; armature of ENP2 comprising one outer spine being plumose proximally and pinnate subdistally, two plumose distal setae and three plumose inner setae; two subdistal inner setae ornamented additional outer spinular ornamentation.

***P4*** (Fig. 2C) as in P2 and P3 except for setal armatures and ornamentations. Coxa with one row of anterior spinules. Outer seta on basis bare and smaller than those of P2 and P3. EXP1 ornamented with six rows of outer spinules; EXP3 with same setal armature of P2 EXP3, but inner seta extremely long, ~2.6× as long as EXP3. Endopod also slightly less than distal end of EXP2; ENP1 lacking anterior tube pore; ENP2 ~2.8× as long as ENP1, ornamented with setules in proximal half on both sides, one row of outer spinules in distal half; anterior tube pore present; setal armature of ENP2 as in P2, but outer seta exceeding distal end of EXP3; spinular ornamentation observed in outer seta and two inner setae.

***P5*** (Fig. 2D). BENP elongate, ~3.3× as long as wide, with one anterior tube pore; outer setophore distinctly prolonged, with one long bare seta, and ornamented with spinules anteriorly and posteriorly; endopodal lobe well-developed, beyond midlength of exopod, slightly tapering towards distal end, with one tube pore, three long pinnate inner setae and two small pinnate distal setae; all margins of endopodal lobe ornamented with spinules. Exopod elongate, ~4.8× as long as wide; distal margin produced as tubular extension, with one long bare seta; inner margin ornamented with spinules and subdistal pinnate seta; outer margin ornamented with three groups of spinules and four pinnate setae; proximal two setae on outer margin displaced in same insertion position.

**Male** (based on paratype MABIK CR00259490). Habitus (Fig. 5A) slightly smaller than female. Body length ranged from 440 to 462 μm (mean = 448.8 μm, *n* = 5); length/width ratio ~4.3 and prosome/urosome (including caudal rami) length ratio 1.0. Urosome (Figs 5A, 6A) six-segmented; P5 bearing-somite trapezoidal in dorsal aspect; genital somite free, slightly broadening towards posterior margin; first and second postgenital somites as in female, with two pairs and one pair of ventral tube pores, respectively.

***Antennule*** (Fig. 5B) seven-segmented, subchirocerate, with geniculation between fifth and sixth segments; first segment armed with one row of weak spinules and two rows of stout spinules; second segment longest, 2.3× as long as wide, subdistally with incomplete suture line; fourth segment represented by small sclerite bearing one seta; fifth segment swollen, with peduncle bearing one long seta and aesthetasc fused basally to adjacent another seta; sixth segment with one bare seta and three teeth-like projections; seventh segment conical, slightly recurved, subdistally with one acrothek as female. Armature formula as follows: 1-[1], 2-[9], 3-[6], 4-[1], 5-[10 + (1 + ae)], 6-[1 + 3 teeth], 7-[8 + acrothek]. All setae bare except for one spinulose seta on second segment and uni-serrate seta on fifth segment; six outer setae on distal segment bi-articulate.

***P3*** (Fig. 6B, B’). Endopod probably 3-segmented, with incomplete anterior division between distal two segments; ENP1 as in that of female; ENP2 with one recurved and pinnate outer spine and one short and plumose inner seta; ENP3 with two inner and two distal plumose setae.

***P5*** (Fig. 6C) two-segmented; left and right BENP fused to sclerite; BENP with one anterior and two inner tube pores; outer setophore cylindrical, flanked with one row of anterior spinules, with one long bare seta. Exopod elongate, 2.3× as long as wide, ornamented with anterior spinules; with two outer, one distal, and two inner setae.

***P6*** (Fig. 6A). asymmetrical, right membrane basally articulated from ventral surface of genital somite; leg represented by small cylindrical protrusion bearing one plumose seta.

##### Etymology.

The species name is derived from the Latin adjective *calidus* meaning ‘warm’ or ‘hot’, and refers the hydrothermal habitat of its type locality. Gender: feminine.

## ﻿Discussion

Huys and Lee (2000) reviewed the taxonomic status of genus *Esola* and several related species, and proposed the subdivision of family Laophontidae into two subfamilies: Laophontinae and Esolinae. The monophyly of the latter subfamily is well supported by two synapomorphies: the superimposed insertion of two proximal outer setae in the female P5, and the male antennule possessing only two segments distal to geniculation. Additionally, the presence of the outer element on the P2 ENP2 and possession of the most proximal seta on the P3 ENP2 in the male are considered symplesiomorphies of the subfamily (Huys and Lee 2000). Based on these contemporary standards, there is little doubt that *Parabathyesola calida* gen. et sp. nov. discovered from the hydrothermal ecosystem in the Onnuri Vent Field should be assigned to subfamily Esolinae. However, its precise taxonomic status remains unclear, and it cannot be assigned confidently to the existing eight genera in the subfamily Esolinae according to the criteria for generic delimitation given by Huys and Lee (2000).

Huys and Lee (2000) also outlined the phylogenetic relationships among the known genera in the subfamily Esolinae using 25 morphological characters. Based on this evidence, the new species collected from the Onnuri Vent Field appears to be closely related to *Bathyesola*, as they share two characters: the absence of an inner seta on P1 ENP1 (derived status) and the presence of five setae on the female P5 (primitive status). Several additional morphological features common to both *Bathyesola* species and the new species further support this suggestion: a trifid compound element (acrothek) composed of one aesthetasc and two setae is present on the antennular segment 4 in females and segment 5 in males; the antennular segment 2 lacks the spinous process in both sexes; the maxillulary endopod has only two distal setae; the maxillipedal syncoxa has only two setae; the P1 EXP3 has two outer spines and two geniculate distal setae; the P3–P4 EXP3 bear only one inner seta; and the male P5 lacks setae. However, this hydrothermal species from the Onnuri Vent Field cannot be assigned to *Bathyesola* due to two significant differences: the six-segmented antennule in the female (vs. seven-segmented in *Bathyesola*) and the presence of an inner seta on P3–P4 ENP1 (vs. absent in *Bathyesola*). These features are considered key diagnostic characters for genera of the Esolinae given by Huys and Lee (2000). Within the subfamily, the loss of an inner seta on the P3 ENP1 is a character unique to *Bathyesola* and *Esola vervoorti* Huys & Lee, 2000. In addition, we did not detect the cup-shaped integumental secretory pores, which probably evolved from a surface precursor pore and occur in all harpacticoids in the subfamily Esolinae except *Archesola* and *Archilaophonte* (Huys and Lee 2000), from our hydrobenthic copepod; these cup-shaped pores are present on the antero-dorsal margin of the cephalothorax in *Bathyesola*. This absence may be the result of secondary loss. Therefore, we propose a new genus, *Parabathyesola* gen. nov., to accommodate *P. calida* gen. et sp. nov. from the Indian hydrothermal vent.

For morphological cladistic analysis, Huys (1990) emphasized the importance of information on sexual dimorphism in the thoracic legs, and showed that an outer element of the female P3 ENP2 is modified as an apophysis in the males of most laophontid harpacticoids, including members of subfamily Esolinae. However, the element of *P. calida* gen. nov. differs from the typical form of family Laophontidae members, possessing a separate, distinct outer spine on the male ENP2 and an incomplete posterior suture (Fig. 6B). Similar atypical features are observed in males of *Archesola typhlops* (Sars, 1908), in which the two-segmented male P3 endopod is as in the female, with an outer spine fused basally to the supporting sediment, except for a reduction in size, instead of the apophysis. Huys and Lee (2000) concluded that this condition may originate during neotenic development in the final molting process. Unfortunately, its lack on male specimens of *Bathyesola compacta*, which is the most closely related species to the novel hydrothermal taxon, led to us limit comparisons on this sexual dimorphic feature between genus *Bathyesola* and *Parabathyesola* gen. nov. The restrained condition of the outer spine as a separate spine can be considered an autapomorphy for the new genus. Furthermore, *Parabathyesola* gen. nov. exhibits an advanced condition of the male P6, characterized by the presence of a single seta, similar to those in genera *Archilaophonte* and *Applanola*, in contrast to the primitive condition of having two setae.

Harpacticoids are the second most abundant and diverse taxon in deep-sea meiofaunal communities (Gomez and Rivera-Sánchez 2021). However, colonization by members of family Laophontidae has generally been unsuccessful (Lee and Huys 1999). They typically occur in comparatively low abundance (<5%) in deep-sea harpacticoid assemblages, such as those at the Anaximenes Seamount in the eastern Mediterranean Sea (George et al. 2018) and in cold-water coral assemblages of the Porcupine Seabight in the northeastern Atlantic (Gheerardyn et al. 2009; George et al. 2018). Notably, laophontids have rarely been found on abyssal plains or in hadal trenches such as the Gulf of California (Gómez and Morales-Serna, 2012) and the Ryukyu and Kuril trenches (Kitahashi et al. 2012, 2013, 2014). Although the species diversity of deep-sea harpacticoids remains poorly studied (Seifried 2004), laophontids appear to have a distributional upper limit extending into abyssal and hadal zones. Nevertheless, a few deep-sea laophontid species have been reported: Laophontidae gen. 1. sp. and Laophontidae gen. 2. sp. from the Anaximenes Seamount (depths of 675–1262 m) in the eastern Mediterranean Sea (George et al. 2018); *Bathylaophonte* species from a hydrothermal vent (depth of 2480 m) on the East Pacific Rise (Nikolov, 2011); Laophontidae gen. 1 sp., *Hololaophonte* (*Hoplolaophonte*?) sp. 1, *Klieonychocamptus* sp. 1, *Laophonte* sp. 1, *Laophonte* sp. 2, and *Paralaophonte* sp. 2 from the Great Meteor Seamount (depths of 287–316 m) in the northeastern Atlantic Ocean (Richter and George 2019); and Laophontidae 1 species from the Gulf of Mexico (Gómez and Rivera-Sánchez 2021).

### ﻿An updated key to genera of the subfamily Esolinae (modified from Huys and Lee 2000)

**Table:** 

1	P2 endopod absent; P2–P4 EXP2 without inner seta; inner distal element on P2–P4 EXP3 delicate and shorter than outer one; P4 ENP2 without inner seta	***Mourephonte* Jakobi, 1953**
–	P2 endopod present; P2–P4 EXP2 with inner seta; inner distal element on P2–P4 EXP3 spinous and longer than outer one; P4 ENP2 with inner seta	**2**
2	Segment 2 of antennule with large outer process; P2 ENP2 with 1 inner seta; male P5 with 2 setae	***Archilaophonte* Willen, 1995**
–	Segment 2 of antennule without spinose outer process; P2 ENP2 with 2 inner setae; male P5 without setae	**3**
3	P1 ENP1 with inner seta; male P3 endopod 2-segmented	***Archesola* Huys & Lee, 2000**
–	P1 ENP1 without inner seta; male P3 endopod 3-segmented	**4**
4	P3 ENP2 with 2 inner setae in females and lacks any seta in males	***Troglophonte* Huys & Lee, 2000**
–	P3 ENP2 with 3 inner setae in females and with 1 inner seta in males	**5**
5	Body depressed dorso-ventrally; P2 ENP2 lacks outer spine	***Applanola* Huys & Lee, 2000**
–	Body cylindrical; P2 ENP2 with outer spine	**6**
6	Female P5 with 3 or 4 setae; caudal rami dorsally with bulbous swelling in females	***Esola* Edwards, 1891**
–	Female P5 BENP with 5 setae; caudal rami not sexually dimorphic	**7**
7	P3–P4 EXP3 with 2 inner setae; distal inner seta on P4 ENP2 transformed	***Corbulaseta* Huys & Lee, 2000**
–	P3–P4 EXP3 with 1 inner seta; distal inner seta on P4 ENP2 not transformed	**8**
8	Female antennule 6-segmented; P3–P4 ENP1 with inner seta	***Parabathyesola* gen. nov.**
–	Female antennule 7-segmented; P3–P4 ENP1 without inner seta	***Bathyesola* Huys & Lee, 2000**

## Supplementary Material

XML Treatment for Parabathyesola

XML Treatment for Parabathyesola calida

## ﻿Additional information

### Use of AI

No use of AI was reported.

### Author ORCIDs

Jong Guk Kim https://orcid.org/0000-0001-5299-9838

Il-Hoi Kim https://orcid.org/0000-0002-7332-0043

Jimin Lee https://orcid.org/0000-0001-9004-8275

### Data availability

All of the data that support the findings of this study are available in the main text.
